# The prevalence of depression and anxiety among Chinese adults with cancer: a systematic review and meta-analysis

**DOI:** 10.1186/1471-2407-13-393

**Published:** 2013-08-22

**Authors:** Yi-Long Yang, Li Liu, Yang Wang, Hui Wu, Xiao-Shi Yang, Jia-Na Wang, Lie Wang

**Affiliations:** 1 Department of Social Medicine, School of Public Health, China Medical University, 92 North 2nd Road, Heping District, Shenyang 110001, People’s Republic of China

## Abstract

**Background:**

A lot of empirical studies have been conducted to evaluate the prevalence of depression and anxiety among Chinese adults with cancer. We aimed to conduct a meta-analysis in order to evaluate the prevalence and odds ratios of depression and anxiety in Chinese adults with cancer compared with those without.

**Methods:**

The three most comprehensive computerized Chinese academic databases-CNKI, Wangfang and Vip databases-were systematically screened through September 2012. PubMed and Web of Science (SCIE) were also searched from their inception until September 2012 without language restrictions, and an internet search was also used. Case–control studies assessing the prevalence of depression and anxiety among Chinese adults with cancer were analyzed. Study selection and appraisal were conducted independently by three authors. The non-weighted prevalence, pooled random-effects estimates of odds ratio (OR) and 95% confidence intervals (CI) were all calculated.

**Results:**

Seventeen eligible studies with a total of 3497 subjects were included. The prevalence of depression and anxiety were significantly higher in adults with cancer compared with those without (Depression: 54.90% vs. 17.50%, OR = 7.85, 95% CI = 5.56-11.07, P = 0.000; Anxiety: 49.69% vs. 18.37%, OR = 6.46, 95% CI = 4.36-9.55, P = 0.000), the same situation was also observed in subgroup of control groups, assessment methods and cancer types. Although no difference of depression was observed in studies utilizing clinical diagnosis compared with self-report, the OR of anxiety in adults with cancer compared with those without was higher in studies utilizing clinical diagnosis (OR = 8.42, 95% CI = 4.83-14.70) than self-reports (OR = 5.83, 95% CI = 3.64-9.34). The ORs of depression and anxiety in cancer patients compared with disease group (Depression: OR = 6.03, 95% CI = 4.23-8.61; Anxiety: OR = 4.40, 95% CI = 3.05-6.36) were lower than in those compared with normal group (Depression: OR = 13.58, 95% CI = 6.26-29.46; Anxiety: OR = 15.47, 95% CI = 10.00-23.95).

**Conclusions:**

We identified high prevalence rates of depression and anxiety among Chinese adults with cancer. The findings support that the prevalence of depression and anxiety among adults with cancer should receive more attention in Chinese medical settings.

## Background

Depression and anxiety are psychological and physiological states characterized by a collection of physical, emotional, and behavioral components [1,2]. They are common psychological disorders that can impair health-related quality of life (including physical, emotional and social dysfunction), significantly increase mortality rate and lead to a massive medical costs [3-6].

Cancer is considered as a serious and potentially life-threatening illness, and even as deadly diseases without treatment (such as some advanced cancers), which has an effect on psychological and physiological states of patients. Unsurprisingly, various studies have demonstrated the high levels of depression and anxiety in cancer patients using a variety of assessment methods. Based on foreign reviews, which mainly included the studies from developed countries like America and UK, the prevalence of major depression and depressive symptoms in cancer patients were 0%-38% and 4.5%-58% respectively [7-10]. The prevalence of anxiety varied from 0.9% to 49% in one review of 58 studies [10], and the range was narrower (5.1%-23%) in large studies using standardized psychiatric interviews [7,11]. In China, the prevalence of depression and anxiety in cancer patients were 25.8%-58% and 32%-40% respectively [12-14].

Cancer patients might be vulnerable to depression and anxiety for many reasons: reactions to cancer diagnosis, the presence of unpleasant symptoms associated with cancer (such as pain, nausea and fatigue), and concerns about disease recurrence or progression. Besides, the physiologic effects of certain treatments (such as high-dose interferon therapy, radiotherapy and chemotherapy) also influenced anxiety and depression [15,16]. Cancer patients with depression may present with worthlessness, hopelessness, lose of energy and interest and suicidal preoccupation [17,18]. And many cancer patients are also anxious, because anxiety is a response to a threat like cancer [19,20], and anxiety has been shown to frequently coexist with depression [17,21]. Sometimes anxiety and depression after cancer diagnosis are adaptive, and may not present a problem. However, some patients continue to have high levels of depression and anxiety that persist for weeks or months, and the untreated anxiety and depression can lead to difficulty with symptom control, hampered treatment decision-making, poor compliance with treatment, prolonged recovery times and impaired quality of life [9,18,22,23].

Nevertheless, evidence is accumulating to suggest that identification and treatment of depression and anxiety among cancer patients will result in reduction in disease progression, improvement in survival rates, reduction in medical costs and improvement in quality of life [22,24,25]. Two recent meta-analyses suggested that compared with control group, psychological intervention effectively improved physical and mental condition of Chinese cancer patients [26,27]. Likewise, some systematic reviews suggested that psychological interventions, like cognitive behavioral therapy (CBT), could be effective against anxiety and depression in cancer patients and have good potential for dissemination in routine clinical practice in America [28,29]. Psychosocial interventions to treat depression and anxiety were also effective even in patients with advanced cancer [29,30].

It should be noted that before antidepressant/anxiolytic medication, and psychotherapy are performed for cancer patients with psychological disorders, the initial recommendation is for evaluation, diagnostic studies, and correction of factors potentially contributing to psychological disorders [29]. Subsequently, effective interventions and special optimum care could be developed for cancer patients based on these findings. Consequently, the first thing we will do is to evaluate the overall prevalence of depression and anxiety in Chinese adults with cancer before planning treatment provision. Although there are many studies evaluating the level of depression and anxiety in Chinese cancer patients, there are some gaps in literatures. First, some studies did not use a control group. We cannot know the level of depression and anxiety of cancer patients compared with other populations. Second, sample size of individual study assessing psychological distress in cancer patients is usually small. Last, a recent Chinese study used the data from 36 cancer registry sites in China and from Third Chinese Death Cause Survey (accepted by GLOBOCAN 2008) to estimate the incidence and mortality rates of cancers in 2008. The numbers of new cases and deaths from cancer was 2.82 million (22.3% of world total) and 1.96 million (25.9%) in China in 2008, and the number will forecast to hit 2.99 million and 2.07 million by 2010, 3.88 million and 2.76 million by 2020, and 4.87 million and 3.60 million by 2030 [31]. Now there has not been a quantitative review, namely meta-analysis, to assess the prevalence of depression and anxiety in Chinese adults with cancer compared with those without, and this situation is similar to foreign countries. Many foreign reviews of cancer patients with psychological distress were only the qualitative literature reviews [9,32,33] or the included studies of the meta-analysis did not use control group as comparison [7].

Therefore, the present meta-analysis aims to synthesize individual study evaluating depression and anxiety in Chinese adults with cancer, and to assess the prevalence and odds ratio (OR) of depression and anxiety in Chinese adults with cancer compared with those without.

## Methods

### Literature search

A systematic search was conducted to identify published literature on the prevalence of depression and anxiety in Chinese adults with cancer. The CNKI database (China National Knowledge Infrastructure), Wanfang database, and Vip database, which are the three most comprehensive Chinese academic database, were searched from their inception until September 2012. We used ‘depression or depressive disorders or depressive symptoms’ and ‘anxiety or anxiety disorder or anxiety symptoms’ combined with ‘cancer or oncology or malignant neoplasm or malignant tumour’ as search themes in the article titles, abstracts and keywords. The reference lists of relevant articles obtained were also screened.

In order to expand searches, PubMed and Web of Science (SCIE) were searched from their inception until September 2012 without language restrictions, and an internet search was also used (e.g., http://www.google.com). The search strategy was: (neoplasms[MeSH Terms] OR cancer[Title/Abstract] OR neoplasms[Title/Abstract] OR oncology[Title/Abstract]) AND (China[MeSH] OR China [Title/Abstract] or Mainland China[Title/Abstract]) AND (depression [MeSH] OR depressive disorder [MeSH] OR depression[Title/Abstract] OR depressive disorder[Title/Abstract] OR depressive symptoms[Title/Abstract] OR anxiety[MeSH] OR anxiety disorders[MeSH] OR anxiety[Title/Abstract] OR anxiety disorders[Title/Abstract] OR anxiety symptoms[Title/Abstract]).

The screening of the abstracts/titles and full-text articles were performed twice by three authors (YLY, LL and YW) independently to reduce reviewer bias and errors.

### Inclusion and exclusion criteria

We included all studies in which: (1) the subjects were aged 18 or older; (2) the subjects of cancer group were patients diagnosed with cancer; (3) case–control studies were eligible, including cancer group and non-cancer control group; (4) studies were included to those involving more than 30 adults with cancer; (5) the subjects had a depression and anxiety according to clinical diagnosis as described in DSM-IV (Diagnostic and Statistical Manual of Mental Disorders, Fourth Edition) [34] or CCMD (Chinese Classification of Mental Disorders) [35] or HRSD/HRSA (Hamilton Rating Scale for Depression and Hamilton Rating Scale for Anxiety) [36,37], or the depression and anxiety of both cancer group and control group were identified by self-report questionnaires that previous studies have established the reliability of them as a measure of depression and anxiety at home and abroad; (6) the prevalence of depression and anxiety were both reported in cancer group and control group; (7) the subjects were from Mainland China (Hong Kong and Macao were excluded due to the long-term European influence). We excluded studies in which: (1) the studies only included cancer patients; (2) it was not sure if the control group excluded the cancer patients; (3) depression and anxiety were measured with the self-edited scales in China that are not widely used and accepted at home and abroad. Eligibility judgment and data extraction were recorded and carried out independently by two authors (LL and YW) in a standardized manner. Any disagreements with them were resolved by discussion and the involvement of another author (LW).

### Quality assessment

Although the existing checklists and quality assessment scales in observational studies is controversial [38], the Newcastle-Ottawa Scale for assessing quality of observational and nonrandomized studies was adapted for use [39]. The instrument evaluated observational studies based on three criteria: selection of cases, comparability of study groups and assessment of outcome or exposure. We defined three categories: the study was considered to have high quality (low risk of bias) if it scored seven points or above, studies that scored 1 or zero for selection or zero for comparability or for assessment of outcome or exposure were categorized as having low quality (high risk of bias), studies that scored in between were considered as having medium quality (moderate risk of bias). Any disagreements with raters (LL and YW) were resolved by discussion and the involvement of another author (LW).

### Meta-analysis

#### Assessment of overall effect size

The effect size of OR is defined as the ratio of odds (odds = Probability/(1-probability) of depression and anxiety occurring in cancer group compared with non-cancer group. An OR greater than 1 indicates that depression/anxiety is more likely to occur in cancer group compared with control group, while an OR less than 1 indicates that the depression/anxiety is less likely to occur in cancer group. The pooled random-effects estimates of OR and 95% confidence intervals (CI) were calculated by standard methods using the inverse variance weighting method, ensuring that the larger more precise estimates were given relatively more weighting, and non-weighted prevalence rates were also calculated. A random effects model was used because it involves the assumption of statistical heterogeneity between studies [40,41]. For zero cell counts, the standard method of adding 0.5 to each cell count was used [42]. Overall effects were analyzed using the statistical software Stata v11.0.

#### Assessment of heterogeneity

Heterogeneity was evaluated with the Q statistic and I^2^ statistic. The Q statistic is used to assess whether differences in results are compatible with chance alone. If the p value of Q statistic is above 0.05, it indicates that there is no significant heterogeneity [43], but the Q statistic is sensitive to the number of studies [44]. To complement the Q statistics, the I^2^ statistic which denotes the variance among studies as a proportion of the total variance was also calculated and reported, because I^2^ is not sensitive to the number of studies [44]. Larger values of I^2^ show increasing heterogeneity. An I^2^ of 0% shows no observed heterogeneity, while 25% shows low, 50% moderate, and 75% high levels of heterogeneity [45].

#### Subgroup analyses

When the hypothesis of homogeneity was rejected by the Q statistic and I^2^ statistic, subgroup analysis was conducted in order to explore potential moderating factors for heterogeneity [44]. Meanwhile, some studies in our meta-analysis included multiple groups (e.g. liver cancer patients and breast cancer patients were compared with a single control group). Subgroup analysis was also used to make sure that each patient was included only once in different subgroups. In our study, subgroup analyses were conducted for moderating factors, including control groups’ type (disease control vs. normal control), assessment methods of depression/anxiety (clinical diagnosis vs. self-report questionnaire) and cancer types. However, due to a few of studies (the number is less than or equal to 3) separately reporting the OR for depression and anxiety in patients with breast cancer, lung cancer, liver cancer, the subgroup comparison of depression and anxiety in different types of cancer patients were not analyzed.

#### Assessment of publication bias

The potential of publication bias of the included studies was first examined by funnel plot symmetry. A funnel plot is a useful graph designed to check the existence of publication bias in meta-analyses. A symmetric funnel shape indicates that publication bias is unlikely, but an asymmetric funnel suggests the possibility of publication bias [46]. However, some authors have argued that visual interpretation of funnel plots is too subjective to be useful [47]. Then Begg’s test and Egger’s test were further used to more objectively test for its presence (as implemented in Stata v11) [48,49].

## Results

### Study selection

**Figure 1:**
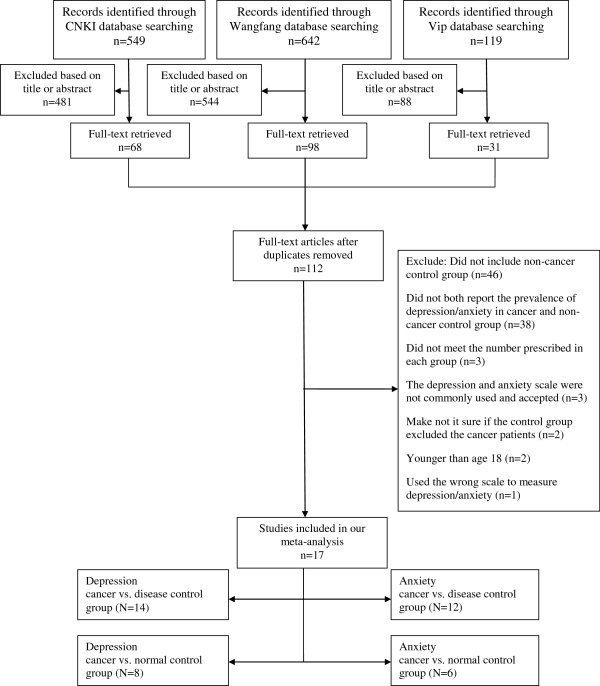
Selection process of studies for the review (Chinese databases).

A flowchart describing the inclusion and exclusion process is presented. As shown in Figure 1, we identified the possibly eligible articles through CNKI database (n = 549), Wangfang database (n = 642) and Vip database (n = 119). The titles and abstracts of these possibly eligible papers were respectively studied by the three authors (YLY, LL and YW), and the full-text articles without duplicates (n = 112) were selected for further examination. Based on the full-text of these 112 studies we finally selected 17 studies for the present meta-analysis [50-66]. The most important reasons for exclusion were: did not include non-cancer control group (n = 46), did not both report the prevalence of depression/anxiety in cancer and non-cancer control group (n = 38). Other reasons included the simple size, the age of subjects, methods of depression and anxiety assessment, and the composition of control group.

**Figure 2:**
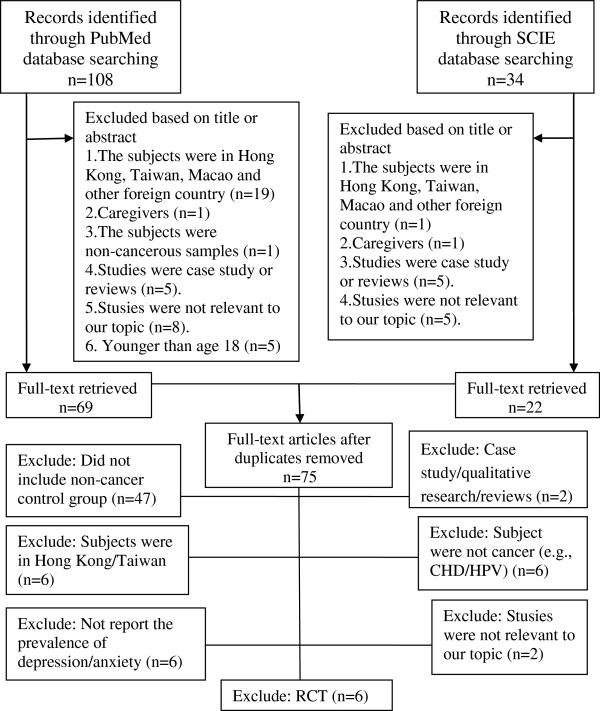
Selection process of studies for the review (international databases).

In order to expand searches, we also searched the international databases of PubMed, SCIE (as shown in Figure 2), and an internet search (e.g., http://www.google.com). However, we did not find any literatures that met our inclusion and exclusion criteria through the international databases search.

### Characteristics of included studies

Due to the different types of control groups, the 17 studies with a total of 3497 subjects produced four subgroups: (1) depression in cancer vs. disease control group (N = 14); (2) depression in cancer vs. normal control group (N = 8); (3) anxiety in cancer vs. disease control group (N = 12); (4) anxiety in cancer vs. normal control group (N = 6) (Figure 1). Study characteristics were listed in Table 1. The studies of this meta-analysis, including 15 journal articles and 2 master’s theses, were published from 2001 to 2010, except for one in 1989. Of the 17 studies three were conducted among breast cancer patients, three among liver cancer patients (one study included both breast cancer and liver cancer patients), two among lung cancer patients, one among esophageal cancer, one among nasopharynx and liver cancer patients, and other studies among different types of cancers. In all of these studies, in addition to one study of primary liver cancer diagnosed by specialist physician [58], different types of cancer were confirmed by the physicians on the basis of cytologic and pathological diagnosis. Regarding to the disease control group, chronic hepatitis [56,58], diabetes [63], tuberculosis [51], benign tumor [62], and other non-cancer medical patients [50,52,54,55,57,60,61,65] were included. Finally, the levels of depression and anxiety were assessed by clinical diagnosis method in five studies [50,52,53,64,66], while that of the other twelve studies was assessed by self-report questionnaires like Self-rating Depression Scale (SDS) and Self-rating Anxiety Scale (SAS).

**Table 1:** Characteristics of the included studies

**Author &Years**	**Depression/Anxiety**	**Participants**	**Mean age**	**Age range**	**Depression/Anxiety**	**Mean score**	**Type of cancer**	**Type of control**	**Prevalence of depression/anxiety (%)**
		**(cancer, n)**			**assessment method and cut-off**				
		**(control, n)**							
Yang & Bao, 2003	Depression	96	53.67	22–76	self-report	47.96 ± 6.73	Mixed	Disease control	63.5
		96	–	–	(SDS index ≥0.5)	32.32 ± 5.86			17.7
	Anxiety	96	53.67	22–76	self-report	49.23 ± 7.68	Mixed	Disease control	60.4
		96	–	–	(SAS index ≥0.5)	34.06 ± 5.01			13.5
Tang, 2008	Depression	150	67	60–82	self-report	–	Mixed	Disease control	48
		50	–	–	(SDS total score ≥11)	–			16
Long et al., 2008	Depression	46	51	36–63	self-report	–	Mixed	Disease control	63.04
		50	50	30–59	(SCL-90-D mean ≥ 1.5)	–			6
	Anxiety	46	51	36–63	self-report	–	Mixed	Disease control	89.13
		50	50	30–59	(SCL-90-A mean ≥ 1.39)	–			62
Zhang et al., 2008	Depression	60	55.9	>18	self-report	52.70 ± 8.70	Mixed	Disease control	73.3
		60	54.8	>18	(SDS standard score ≥ 50)	43.98 ± 9.35			31.7
	Depression	60	55.9	>18	self-report	52.70 ± 8.70	Mixed	Normal control	73.3
		60	54.8	>18	(SDS standard score ≥ 50)	38.43 ± 7.59			8.3
	Anxiety	60	55.9	>18	self-report	52.95 ± 8.35	Mixed	Disease control	70
		60	54.8	>18	(SAS standard score ≥ 50)	45.82 ± 10.01			48.3
	Anxiety	60	55.9	>18	self-report	52.95 ± 8.35	Mixed	Normal control	70
		60	54.8	>18	(SAS standard score ≥ 50)	35.92 ± 8.04			11.7
Tao et al., 2005	Depression	72	47	21–69	self-report	0.54 ± 0.05	Nasopharynx/liver cancer	Disease control	77.8
		30	43	22–65	(SDS index ≥ 0.51)	0.42 ± 0.06			23.3
	Depression	72	47	21–69	self-report	0.54 ± 0.05	Nasopharynx/liver cancer	Normal control	77.8
		30	42	23–65	(SDS index ≥ 0.51)	0.39 ± 0.05			10
	Anxiety	72	47	21–69	self-report	50 ± 8	Nasopharynx/liver cancer	Disease control	83.3
		30	43	22–65	(SAS standard score ≥ 41)	37 ± 5			40
	Anxiety	72	47	21–69	self-report	50 ± 8	Nasopharynx/liver cancer	Normal control	83.3
		30	42	23–65	(SAS standard score ≥ 41)	32 ± 5			13.3
Liu et al., 2001	Depression	45	59.24	24–78	clinical diagnosis	16.78 ± 7.75	Mixed	Normal control	55.56
		45	–	–	(HRSD total score ≥ 17)	5.87 ± 4.67			4.44
	Anxiety	45	59.24	24–78	clinical diagnosis	14.82 ± 6.51	Mixed	Normal control	46.67
		45	–	–	(HRSA total score ≥ 14)	6.47 ± 4.73			6.67
Zhang et al., 2009	Depression	100	58.86	35–76	clinical diagnosis	42.46 ± 12.74	Breast cancer	Normal control	89
		100	–	–	(HRSD total score ≥ 20)	34.97 ± 8.31			18
	Anxiety	100	58.86	35–76	clinical diagnosis	43.24 ± 10.38	Breast cancer	Normal control	78
		100	–	–	(HRSA total score ≥ 14)	32.25 ± 8.26			22
Wang et al., 2005	Depression	60	58	34–75	self–report	–	Lung cancer	Normal control	39.58
		30	51	26–68	(SDS standard score ≥ 50)	–			10
	Anxiety	60	58	34–75	self-report	–	Lung cancer	Normal control	43.75
		30	51	26–68	(SAS standard score ≥ 50)	–			6.67
Tian et al., 2005	Depression	112	55.3	36–72	self-report	52.21 ± 5.61	Liver cancer	Disease control	53.6
		152	45.6	30–60	(SDS standard score ≥ 50)	44.45 ± 7.66			16.4
	Anxiety	112	55.3	36–72	self-report	51.1 ± 4.64	Liver cancer	Disease control	51.8
		152	45.6	30–60	(SAS standard score ≥ 50)	42.99 ± 7.17			24.3
Liu, 2006	Depression	124	48	18–70	clinical diagnosis	16.95 ± 0.70	Mixed	Disease control	33.26
		60	–	18–70	(HRSD total score ≥ 20)	6.80 ± 1.14			3.33
	Anxiety	124	48	18–70	clinical diagnosis	9.39 ± 0.51	Mixed	Disease control	29.84
		60	–	18–70	(HRSA total score ≥ 17)	6.30 ± 0.83			6.67
	Depression	124	48	18–70	clinical diagnosis	16.95 ± 0.70	Mixed	Normal control	33.26
		60	–	18–70	(HRSD total score ≥ 20)	4.67 ± 0.92			1.67
	Anxiety	124	48	18–70	clinical diagnosis	9.39 ± 0.51	Mixed	Normal control	29.84
		60	–	18–70	(HRSA total score ≥ 17)	3.63 ± 0.67			1.67
Yuan & Zheng, 2004	Depression	30	36.1	34–38	self-report	41.83 ± 12.83	Breast cancer	Disease control	23.3
		30	35.3	25–47	(SDS standard score > 53)	35.63 ± 6.99			0
	Anxiety	30	36.1	34–38	self–report	48.93 ± 13.35	Breast cancer	Disease control	33.3
		30	35.3	25–47	(SAS standard score > 50)	39.30 ± 9.01			3.3
Gao et al., 1989	Depression	245	46.1	20–76	self-report	–	Mixed	Disease control	73.1
		232	46.4	20–74	(CES-D total score ≥ 16)	10.96 ± 6.46			22.5
	Anxiety	245	46.1	20–76	self-report	–	Mixed	Disease control	31.1
		232	46.4	20–74	(STAI total score ≥ 27)	13.92 ± 8.15			4.8
Chen & Gao, 2010	Depression	90	53.5	38–79	clinical diagnosis	–	Esophageal cancer	Disease control	46.7
		86	51.8	36–80	(HRSD total score ≥ 20)	–			12.8
	Anxiety	90	53.5	38–79	clinical diagnosis	–	Esophageal cancer	Disease control	48.9
		86	51.8	36–80	(HRSA total score ≥ 7)	–			17.4
She, 2009	Depression	142	44.53	15–82	self-report	50.85 ± 11.57	Mixed	Disease control	60.6
		149	44.53	15–82	(SDS standard score ≥ 50)	46.09 ± 12.16			38.3
	Anxiety	142	44.53	15–82	self-report	47.80 ± 10.8	Mixed	Disease control	47.2
		149	44.53	15–82	(SAS standard score ≥ 50)	44.51 ± 10.04			23.5
Zhao et al., 2001	Depression	65	51.5	29–71	self-report	0.54 ± 0.08	Liver cancer	Disease control	43.08
		65	–	–	(SDS index ≥0.5)	0.27 ± 0.12			9.23
	Anxiety	65	51.5	29–71	self-report	36.86 ± 6.47	Liver cancer	Disease control	24.62
		65	–	–	(SAS standard score ≥ 50)	27.1 ± 9.76			13.69
	Depression	65	51.5	29–71	self-report	0.41 ± 0.09	Breast cancer	Disease control	20
		65	–	–	(SDS index ≥0.5)	0.27 ± 0.12			9.23
	Anxiety	65	51.5	29–71	self-report	44 ± 8.36	Breast cancer	Disease control	20
		65	–	–	(SAS standard score ≥ 50)	27.1 ± 9.76			13.96
Wan et al., 2004	Depression	100	44.51	20–70	self-report	15.06 ± 11.5	Primary liver cancer	Disease control	49
		100	–	–	(CES-D total score ≥ 16)	11.03 ± 15.06			27
	Depression	100	44.51	20–70	self-report	15.06 ± 11.5	Primary liver cancer	Normal control	49
		100	–	–	(CES-D total score ≥ 16)	8.08 ± 8.44			17
Zhang et al., 2003	Depression	155	–	>18	Clinical diagnosis	21 ± 9	Lung cancer	Normal control	43.2
		155	–	>18	(HRSD total score >8)	9 ± 4			16.1

*Abbreviations: SDS* self-rating depression scale, *SAS* self-rating anxiety scale, *SCL-90-D* symptom checklist 90-depression, *SCL-90-A*, symptom checklist 90-anxiety, *HRSD* hamilton rating scale for depression, *HRSA* hamilton rating scale for anxiety, *CES-D* center for epidemiologic studies depression scale, *STAI* state-trait anxiety inventory; -, no report.

### Risk of bias assessment

Ratings of study quality for each of the Newcastle-Ottawa criteria were presented in Table 2. As shown in Table 2, higher scores reflect the better study quality, and the average scores of all studies were above 5. Seven studies were judged to have low quality for selection of cases or assessment of outcome or exposure and two of high quality; other studies were rated as medium quality.

**Table 2:** Assessment of study quality

**Studies**	**Quality Indicators from Newcastle-Ottawa scale**									
	**1**	**2**	**3**	**4**	**5A**	**5B**	**6**	**7**	**8**	**Total score**
Yang & Bao, 2003	Yes	No	No	Yes	Yes	Yes	No	Yes	No	5
Tang, 2008	Yes	No	No	Yes	Yes	Yes	No	Yes	No	5
Long et al., 2008	Yes	No	No	Yes	Yes	Yes	No	Yes	No	5
Zhang et al., 2008	Yes	No	Yes	Yes	Yes	Yes	No	Yes	No	6
Tao et al., 2005	Yes	No	Yes	Yes	Yes	Yes	No	Yes	No	6
Liu et al., 2001	Yes	No	Yes	Yes	Yes	Yes	Yes	Yes	No	7
Zhang et al., 2009	Yes	No	Yes	Yes	Yes	Yes	No	Yes	No	6
Wang et al., 2005	Yes	No	Yes	Yes	Yes	Yes	No	Yes	No	6
Tian et al., 2005	Yes	No	No	Yes	Yes	Yes	No	Yes	No	5
Liu, 2006	Yes	No	Yes	Yes	Yes	Yes	Yes	Yes	No	7
Yuan & Zheng, 2004	Yes	No	No	Yes	Yes	Yes	No	Yes	No	5
Gao et al., 1989	Yes	No	No	Yes	Yes	Yes	No	Yes	No	5
Chen & Gao, 2010	Yes	No	No	Yes	Yes	Yes	No	Yes	No	5
She, 2009	Yes	No	No	Yes	Yes	Yes	No	Yes	Yes	6
Zhao et al., 2001	Yes	No	No	Yes	Yes	Yes	No	Yes	Yes	6
Wan et al., 2004	Yes	No	Yes	Yes	Yes	Yes	No	Yes	No	6
Zhang et al., 2003	Yes	No	Yes	Yes	Yes	Yes	Yes	Yes	No	7

*Abbreviations*: 1 indicates cases independently validated; 2, cases are representative of population; 3, community controls; 4, controls have no history of cancer; 5A, study controls for age/gender; 5B, study controls for additional factor(s); 6, ascertainment of depression/anxiety by blinded structured interview or secure record; 7, same method of ascertainment used for cases and controls; and 8, nonresponse rate the same for cases and controls.

### Prevalence rates of depression and anxiety in cancer patients

As shown in Table 3, the overall prevalence of depression and anxiety was higher in adults with cancer compared with those without (P < 0.001). This finding was consistent when the prevalence was determined by control groups, method of depression/anxiety assessment and cancer types (P < 0.001).

**Table 3:** Unadjusted prevalence of depression and anxiety in adults with and without cancer

	**No. of studies**	**No. of subjects**	**Cancer subjects (%)**	**Non-cancer subjects (%)**
**Depression** (All)	17	3484	54.90***	17.50
Control group				
Disease control	13	2554	54.84***	19.61
Normal control	8	1286	55.03***	12.98
Method of depression assessment				
Clinical diagnosis	5	1010	47.49***	11.90
Self-report questionnaire	12	2474	58.11***	19.65
Cancer type				
Breast cancer	3	380	55.90***	12.97
Lung cancer	2	400	42.33***	15.14
Liver cancer	3	794	49.34***	17.99
**Anxiety **(All)	14	2684	49.69***	18.37
Control group				
Disease control	11	2154	46.64***	20.30
Normal control	6	786	57.27***	12.00
Method of depression assessment				
Clinical diagnosis	4	650	44.93***	12.82
Self-report questionnaire	10	1974	51.74***	20.27
Cancer type				
Breast cancer	3	390	58.46***	16.41
Lung cancer	1	90	43.33***	6.67
Liver cancer	2	394	41.81***	21.20

*** Prevalence of depression and anxiety significantly greater in patients with cancer compared with a non-cancer control group (P < 0.001).

Note: The No. of studies per row is based on the independent group of cancer vs. control group. However, some studies included multiple control groups (e.g., disease and normal control). Thus, the total No. of studies per subgroup of control group is higher than the total number of the included studies in our meta-analysis.

The overall prevalence of depression and anxiety were 54.6% and 49.69% in Chinese adults with cancer, and the prevalence of depression and anxiety were 18.37% and 17.50% in non-cancer group. This prevalence of depression was higher in studies utilizing self-reports than in studies using clinical diagnosis among cancer patients (58.11% vs. 47.49%, P = 0.000), and the same situation was also observed among control group (19.65% vs. 11.90%, P = 0.000). Meanwhile, the prevalence of anxiety was also higher in self-reports than in clinical diagnosis among cancer patients (51.74% vs. 44.93%, P = 0.012), and the same situation was observed among control group (20.27% vs. 12.82%, P = 0.002).

### Odds ratios of depression and anxiety in cancer patients

**Figure 3:**
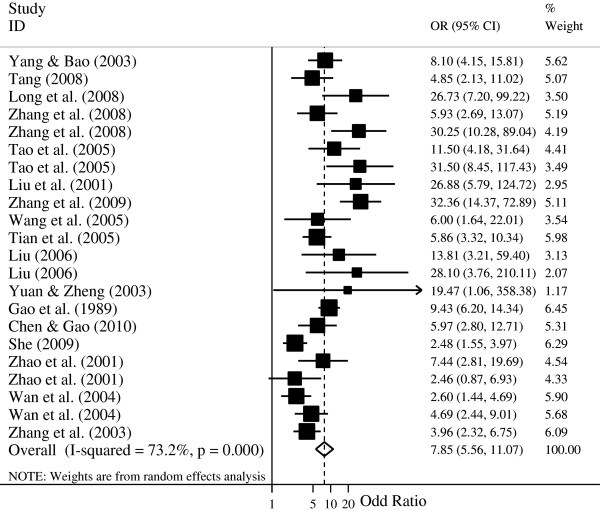
**Forest plot for the meta-analysis of depression in adults with and without cancer.** Note: Some studies included multiple cancer types (e.g., liver and breast cancer) and control groups (e.g., disease and normal control) in our meta-analysis. Thus, the total number of independent group in the forest plot is higher than the total number of the included studies in our meta-analysis.

**Figure 4:**
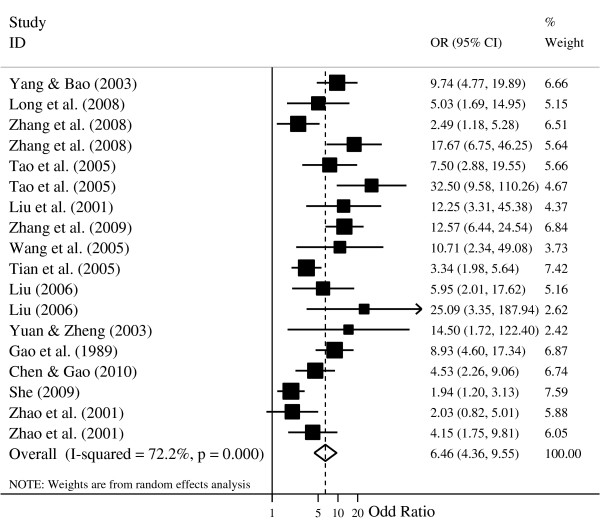
**Forest plot for the meta-analysis of anxiety in adults with and without cancer.** Note: Some studies included multiple cancer types (e.g., liver and breast cancer) and control groups (e.g., disease and normal control) in our meta-analysis. Thus, the total number of independent group in the forest plot is higher than the total number of the included studies in our meta-analysis.

A pooled random effects meta-analysis was conducted using data from 17 studies, which estimated the levels of depression and anxiety in adults with cancer compared with those without. This analysis included data for 1,711 adults with cancer and 1,740 without cancer. As shown in Figures 3 and 4, the odds of depression was associated with a 7.85-fold increased risk of cancer patients when compared with control group (OR = 7.85, 95% CI = 5.58-11.07; p = 0.000), and the odds of anxiety was also more than six times as high in cancer patients compared with control group (OR = 6.46, 95% CI = 4.36-9.55; p = 0.000). However, the heterogeneity analysis of the effect sizes of depression (Q = 78.36, p = 0.000; I^2^ = 73.2%) and anxiety (Q = 61.21, p = 0.000; I^2^ = 72.2%) showed that there was a relatively high amount of heterogeneity in our meta-analysis.

### Subgroup analyses

As shown in Table 4, the ORs of depression and anxiety were significantly increased in adults with cancer compared with those without on moderating factors, including the subgroup of control groups, assessment methods of depression/anxiety and cancer types. The ORs of depression and anxiety in cancer patients compared with disease control group (Depression: OR = 6.03, 95% CI = 4.23-8.61, I^2^ = 65.5%; Anxiety: OR = 4.40, 95% CI = 3.05-6.36, I^2^ = 61.6%) were lower than in those compared with normal control group (Depression: OR = 13.58, 95% CI = 6.26-29.46, I^2^ = 79.7%; Anxiety: OR = 15.47, 95% CI = 10.00-23.95, I^2^ = 0%).

**Table 4:** Odds ratios of depression and anxiety in adults with and without cancer: subgroup analyses

**Subgroups**	**No. of studies**	**No. of subjects**	**OR**	**95% CI**	**Q**	**I**^**2 **^**(%)**	**P**^**§**^
**Depression**							
Control group							0.013
Disease control	13	2554	6.03	4.23-8.61	37.63***	65.5	
Normal control	8	1286	13.58	6.26-29.46	34.56***	79.7	
Method of depression assessment							0.094
Clinical diagnosis	5	1010	12.41	5.16-29.84	23.33***	78.6	
Self-report questionnaire	12	2474	6.83	4.70-9.93	52.23***	71.3	
Cancer type							-
Breast cancer	3	380	10.84	1.44-81.85	14.83***	86.5	
Liver cancer	3	794	4.54	2.92-7.05	5.20	42.3	
Lung cancer	2	400	4.20	2.57-6.88	0.34	0	
**Anxiety**							
Control group							0.000
Disease control	11	2154	4.40	3.05-6.36	28.62**	61.6	
Normal control	6	786	15.47	10.00-23.95	2.44	0	
Method of anxiety assessment							0.013
Clinical diagnosis	4	650	8.42	4.83-14.70	6.31	36.6	
Self-report questionnaire	10	1974	5.83	3.64-9.34	48.78***	75.4	
Cancer type							-
Breast cancer	3	390	8.24	3.49-19.44	4.22	52.6	
Liver cancer	2	394	2.95	1.87-4.63	0.87	0	

**p < 0.01.

***p < 0.001.

^§^ P of comparison between these subgroups.

Note: The No. of studies per row is based on the independent group of cancer vs. control group. However, some studies included multiple control groups (e.g., disease and normal control). Thus, the total No. of studies per subgroup of control group is higher than the total number of the included studies in our meta-analysis. -, no report.

ORs were also obtained for studies using different methods of depression and anxiety assessment. Although no difference of depression was observed in studies utilizing clinical diagnosis compared with self-report, a significant smaller OR of anxiety was observed in studies utilizing self-reports (OR = 5.83, 95% CI = 3.64-9.34, I^2^ = 75.4%) compared with clinical diagnosis (OR = 8.42, 95% CI = 4.83-14.70, I^2^ = 36.6%).

Due to the small number of studies, the subgroup comparison of depression and anxiety in different types of cancer patients were not analyzed.

### Publication bias

Visual inspection of the funnel plot indicated some publication bias, and the Begg’s test and Egger’s test further suggested publication bias in depression (Begg’s test, P = 0.021; Egger’s test, P = 0.019) and anxiety (Begg’s test, P = 0.15; Egger’s test, P = 0.017) in our meta-analysis.

## Discussion

At the beginning of discussion, we would assess the heterogeneity and study quality in the present meta-analysis. First, we performed strict inclusion criteria, random effects models and subgroup analyses to control and reduce the heterogeneity. However, the heterogeneity was still relatively higher, and the conclusion should be considered with some caution. Second, the Newcastle-Ottawa Scale was used to assess the study quality. We only identified two high-quality studies. The bias of medium-quality and low-quality studies mainly included selection of cases and assessment of outcome or exposure. Quality assessment indicated some methodological weaknesses, which could weaken the internal validity.

The overall prevalence of depression and anxiety in Chinese patients with cancer were 54.9% (range: 20%-89%) and 49.69% (range: 20%-89.13%) in our meta-analysis, suggesting that depression and anxiety also did coexist in Chinese cancer patients, similar to this situation in foreign countries [17,21]. This situation should be noticed because comorbid anxiety and depressive disorders tend to have severe symptoms, poorer outcomes and greater use of healthcare resources than those with a single disorder [67]. Meanwhile, the results were slightly higher than some empirical studies assessing the prevalence of depression and anxiety in China [12-14].

We also compared our results with other four relatively comprehensive reviews of depression/anxiety in cancer patients: (1) the review of Pirl reported the prevalence of depression (10%-25%) based on 350 English-language literatures published between 1966 and 2001 [68]; (2) Hotopf showed the prevalence of depression for self-reporting questionnaire (29%) and clinical diagnosis (15%) based on 46 literatures published before 2000 [9]; (3) Van’t Spijker indicated the prevalence of depression (0%-46%) and anxiety (0.9%-49%) from 58 studies published from 1980 to 1994 [10]; (4) Mitchell reported the prevalence of depression (20.7%-24.6%) and anxiety (9.8%-10.3%) of 94 interview-based studies published before 2010 [7]. There might be several reasons for the different prevalence. First explanation might be that we only identified 17 eligible studies, but other reviews included at least 46 studies. Our results may be overestimated due to the little studies and high data fluctuation. Second explanation might be that most of the included studies of these reviews are from developed countries which have lower prevalence of mental health problems as compared to developing countries like China [69]. Third explanation might be that depression and anxiety in cancer patients were assessed using different questionnaires. The included studies of these reviews mainly used the Hospital Anxiety and Depression Scale (HADS), but SDS/SAS was the most commonly used in our meta-analysis. Last explanation might be that many studies of these reviews used clinical diagnosis like HRSD/HRSA, but only five studies of our meta-analysis employed clinical diagnosis method. Some studies indicated that the prevalence of depression and anxiety would be overestimate by self-report questionnaires compared with standardized clinical diagnoses [20,70].

Only reporting the prevalence of depression and anxiety in Chinese cancer patients is not enough, it is important that comparable control groups are involved so that the level of depression and anxiety in cancer patients can be reliably and accurately determined. The level of depression (OR = 7.85, 95% CI = 5.56-11.07) and anxiety (OR = 6.46, 95% CI = 4.36-9.55) were significantly higher in adults with cancer compared with those without. This is the first meta-analysis reporting depression and anxiety in Chinese cancer patients compared with those without. A meta-analytical review also reported depression and anxiety in cancer patients compared with non-cancer group [10], but it yielded an overall effect size of d-value (mean difference), rather than OR/RR.

Through the subgroups analysis of control groups (disease control vs. normal control), cancer patients compared with normal group experienced the higher level of depression/anxiety than them compared with disease group at the 0.05 significance level. More importantly, cancer patients were significantly more depressed and anxious when compared with normal and disease groups, respectively. The majority of studies mainly focused on the psychological disorders in cancer patients and ignored the role and type of control group [7-9,12-14,68]. The results showed that the odds of depression/anxiety were nearly 4–6 times as high in cancer patients, even when compared with disease control including patients with chronic hepatitis [56,58], diabetes [63], tuberculosis [51], benign tumor [62], and other non-cancer medical patients [50,52,54,55,57,60,61,65]. However, the different level of depression/anxiety between cancer patients and non-cancer populations has been controversial. The study of Reyes-Gibby indicated that respondents with a history of cancer had excess risk for depression (OR = 1.21; 95% CI = 1.06-1.37) compared to those without [71]. But some studies demonstrated that depression was common not only among cancer patients, but also nearly equally among non-cancer diseases [72,73].

Some studies suggested the discrepancy between clinical diagnosis and self-reports measuring depression/anxiety among cancer patients [29,74], and indicated the results would be overestimate by self-report questionnaires compared with clinical diagnoses [20,70]. Our results seemed to be consistent with the conclusion above. The prevalence of depression/anxiety in cancer patients was higher in self-reports compared with clinical diagnosis, and this situation also occurred in non-cancer group. However, when control groups were involved, we found that a significant smaller OR of anxiety was observed in studies utilizing self-reports compared with clinical diagnosis, although not significantly difference between clinical diagnoses and self-reports of depression. This discrepancy may result from the same reason that these studies only focused on the cancer patients and ignored the control group when different assessment methods of depression and anxiety were used [20,29,70,71]. However, only five and four studies with a combined population of 1010 and 650 were conducted to measure depression/anxiety using clinical diagnoses based on HRSD/HRSA, it was necessary to consider that our results might be influenced by small number of studies.

### Implication

There are several theoretical and practical implications through our meta-analysis. In theory, future studies assessing psychological disorders among cancer patients should include and specify control group. Thus, they could explore the difference levels of depression/anxiety between cancer patients and other populations, including other non-cancer diseases, and on the other hand, a whole new perspective would be provided for researchers on the use of self-reports and clinical diagnosis to assess depression/anxiety in cancer patients when control groups were involved. In practice, first, some studies have shown that it is necessary to evaluate the prevalence of depression and anxiety before any effects can be provided for optimum care among cancer patients and reduction of psychological disorders [17,18,29]. The present meta-analysis provided the necessary preparations for oncologists and physicians to treat and manage depression and anxiety in Chinese cancer patients; second, although a brief psychological intervention could promote the quality of life and reduce depression and anxiety among cancer patients [75], oncologists and physicians still remained poor at detecting and treating their cancer patients’ psychological problems [76-78]. There might be many reasons for this situation, but one of these reasons could be that oncologists and physicians were unaware of the levels of depression and anxiety in cancer patients. This meta-analysis showed that cancer patients had higher prevalence of depression and anxiety, indicating that depression and anxiety in Chinese cancer patients should be received sufficient attention.

### Limitation

The present meta-analysis had several limitations. First, although some studies demonstrated that depression and anxiety might play a causal role of cancer [25,79], the present meta-analysis was based on cross-sectional studies, which could not determine the causation or temporality of this association between the development of cancer and depression/anxiety. Second, a lot of studies showed that there was a significant relationship between depression/anxiety in cancer patients and age [18,80], cancer type [10,81,82], gender [83,84], income [85] and so on, but our meta-analysis did not provide enough information and number of studies regarding these potential moderating factors. Third, although we employed subgroup analysis to explore potential sources of heterogeneity including control group, methods of depression/anxiety assessment and cancer types, the subgroup analysis could not reduce I^2^ to 50% or less in many cases. Fourth, only five and four studies with a combined population of 1010 and 650 were conducted using HRSD/HRSA to measure clinically significant anxiety and depression. Thus, depression and anxiety in our meta-analysis more often referred to the depressive symptom and anxiety symptom. Fifth, we did not find any international literatures that meet our inclusion and exclusion criteria. It might be because that the international journals require the papers at a high level compared with Chinese journals, and international journals maybe not receive the simple descriptive research. Sixth, as some studies included multiple groups in the meta-analysis, cancer patients or people in control group were included twice when calculating the overall OR of depression/anxiety. This will overestimate the precision. Finally, the high risk of publication bias is another (and perhaps the most important) limitation.

## Conclusions

We conclude that Chinese adults with cancer had higher prevalence rates of depression and anxiety compared with control group, and the same situation is observed in subgroup analyses. The findings support that the prevalence of depression and anxiety among adults with cancer should receive more attention in Chinese medical settings.

## Competing interests

The authors declare that they have no conflict of interest.

## Authors’ contributions

YLY was responsible for conception and design of the review, carried out the literature search, performed data extraction and data analysis, and wrote the manuscript. LL and YW carried out the literature search, performed data extraction, resolved the disagreement, and participated in conception and design of the review. HW, SXY and JNW participated in conception and design of the review, and critically revised the manuscript. LW supervised the data collection, statistical analysis and paper writing. All authors read and approved the final manuscript.

## Pre-publication history

The pre-publication history for this paper can be accessed here:

http://www.biomedcentral.com/1471-2407/13/393/prepub
